# Housing Quality in a Rural and an Urban Settlement in South Africa

**DOI:** 10.3390/ijerph18052240

**Published:** 2021-02-24

**Authors:** Angela Mathee, Jocelyn Moyes, Thulisa Mkhencele, Jackie Kleynhans, Brigitte Language, Stuart Piketh, Elias Moroe, Floidy Wafawanaka, Neil Martinson, Meredith McMorrow, Stefano Tempia, Kathleen Kahn, Cheryl Cohen

**Affiliations:** 1 Environment and Health Research Unit, South African Medical Research Council, Johannesburg 2028, South Africa; 2 Faculty of Health Sciences, School of Public Health, University of the Witwatersrand, Johannesburg 2193, South Africa; cherylc@nicd.ac.za; 3 Environmental Health Department, Faculty of Health Sciences, University of Johannesburg, Johannesburg 2028, South Africa; 4 Center for Respiratory Disease and Meningitis, National Institute for Communicable Diseases, Johannesburg 2193, South Africa; jocelynm@nicd.ac.za (J.M.); thulisam@nicd.ac.za (T.M.); jackiel@nicd.ac.za (J.K.); 5 Unit for Environmental Science and Management, School of Geo- and Spatial Science, North-West University, Potchefstroom 2520, South Africa; brigittelanguage@gmail.com (B.L.); stuart.piketh@nwu.ac.za (S.P.); 6 Perinatal HIV Research Unit (PHRU), University of the Witwatersrand, Johannesburg 1864, South Africa; eliasmoroe@gmail.com (E.M.); martinson@phru.co.za (N.M.); 7 MRC/Wits Rural Public Health and Health Transitions Research Unit (Agincourt), Faculty of Health Sciences, School of Public Health, University of the Witwatersrand, Johannesburg 2193, South Africa; floidy.wafawanaka@wits.ac.za (F.W.); kathleen.kahn@wits.ac.za (K.K.); 8 Influenza Division, National Center for Immunization and Respiratory Diseases, U.S. Centers for Disease Control and Prevention, Atlanta, GA 30329, USA; bwe3@cdc.gov (M.M.); stefanot@nicd.ac.za (S.T.); 9 Influenza Program, Centers for Disease Control and Prevention—South Africa, Pretoria 0181, South Africa; 10 MassGenics, Duluth, GA 30096, USA

**Keywords:** housing, water, air pollution, fuel use, environmental health, South Africa

## Abstract

During 2016 to 2018, a prospective household cohort study of influenza and respiratory syncytial virus community burden and transmission dynamics (the PHIRST study) was undertaken to examine the factors associated with influenza and other respiratory pathogen transmissions in South Africa. We collected information on housing conditions in the PHIRST study sites: Rural villages near Agincourt, Bushbuckridge Municipality, Mpumalanga Province, and urban Jouberton Township in North West Province. Survey data were collected from 159 and 167 study households in Agincourt and Jouberton, respectively. Multiple housing-related health hazards were identified in both sites, but particularly in Agincourt. In Agincourt, 75% (119/159) of households reported daily or weekly interruptions in water supply and 98% (154/159) stored drinking water in miscellaneous containers, compared to 1% (1/167) and 69% (115/167) of households in Jouberton. Fuels other than electricity (such as wood) were mainly used for cooking by 44% (70/159) and 7% (11/167) of Agincourt and Jouberton households, respectively; and 67% (106/159) of homes in Agincourt versus 47% (79/167) in Jouberton were located on unpaved roads, which is associated with the generation of dust and particulate matter. This study has highlighted housing conditions in Agincourt and Jouberton that are detrimental to health, and which may impact disease severity or transmission in South African communities.

## 1. Introduction

The quality of housing, which includes aspects related to shelter, water, fuel use for cooking and space heating, and solid waste management, as well as factors such as crowding, is a potent determinant of health status [1]. The World Health Organization (WHO) estimates that approximately one-quarter of the global burden of disease is due to modifiable environmental factors such as housing conditions; the proportion in children may be as high as one third [2]. In under-developed settings, including parts of South Africa, the housing-related contribution to the global burden of disease may be higher still, and consideration of housing-related factors in disease transmission takes on increasing importance [1]. Water access, quality, and security (a regular, uninterrupted supply), as well as the efficacy of sanitation, solid waste, and wastewater removal services play important roles in the prevention of infectious diseases such as diarrhea and respiratory infections, including influenza [3,4,5]. The type of fuel used for cooking (or water/space heating), together with the quality of cooking and heating appliances, ventilation practices, and overcrowding can influence household exposure to indoor air pollution and the risk of a range of respiratory and communicable diseases [6]. Wood and other biomass have been described as a major source of household air pollution, generating fine particles (around PM_2.5_ or smaller), which are of particular public health concern [7]. A shift from household use of biomass to electricity and other safer fuels is expected to bring about significant improvements in public health [8]. There is however a paucity of detailed information on housing conditions in developing countries, especially in African settings.

In recent years, there has been growing attention to the role of housing factors in the transmission of influenza. In the United States of America, ambient air pollution was linked to influenza and pneumonia [9]. In China, Wang et al. [10] found a dose-response trend in exposure to indoor air pollution and influenza-like illness (ILI) after controlling for potential confounding factors: Cooking frequency, the use of coal as the primary fuel, and ventilation practices were associated with ILI [10]. In a large-scale study (47 cities) undertaken in China, exposure to particulate matter (PM_2.5_) specifically, was significantly associated with influenza [11]. The authors attributed more than 10% of incident influenza cases to exposure to fine particulate matter [11]. In addition, regular handwashing, which is reliant on access to adequate supplies of water, has been associated with a reduced risk of influenza infection [12,13]. In recognition of the important role of housing quality in health, the WHO has published general guidelines on housing and health [1], as well as guidelines focused on specific aspects of the housing environment, such as water quality [14] and household air pollution [7].

During 2016 to 2018, a prospective household cohort study of influenza and respiratory syncytial virus community burden and transmission dynamics (the PHIRST study) was undertaken to examine the factors associated with influenza and other respiratory pathogen transmissions in South Africa. Given the established role of housing conditions in disease transmission, we describe local housing conditions for inclusion in analyses of disease transmission in the PHIRST study sites.

## 2. Materials and Methods

### 2.1. Study Setting and Design

**Figure 1:**
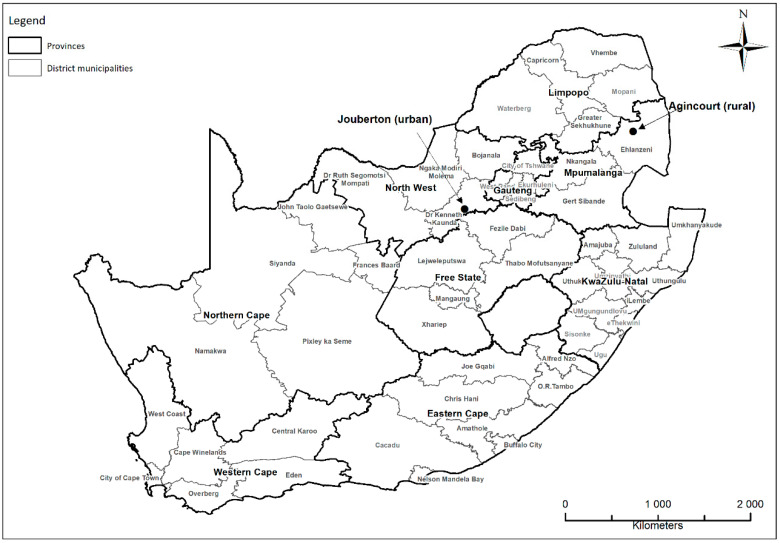
Map of South Africa showing the location of the study sites: PHIRST ^†^, 2016–2018. ^†^ PHIRST: Prospective Household cohort study of Influenza and Respiratory Syncytial virus community burden and Transmission dynamics in South Africa. Map: Thandi Kapwata, SAMRC Environment & Health Research Unit.

From 2016 to 2018, we enrolled cohorts into the PHIRST study in two sites in South Africa: In the Agincourt area of Bushbuckridge Local Municipality, Mpumalanga Province, and in Jouberton Township, City of Klerksdorp, North West Province (see Figure 1). Agincourt is a rural area with communities clustered into villages [15], while Jouberton is a large urban township. In Agincourt, PHIRST was undertaken within the Health and socio-Demographic Surveillance System (HDSS) of the SAMRC/Wits University Rural Public Health and Health Transitions Research Unit, and in Jouberton, the study was locally managed by the Perinatal HIV Research Unit (PHRU) [16]. While a separate sample of households was randomly drawn in Jouberton during each of the three study years (2016 through 2018), in Agincourt two villages were chosen each year, and a random sample of households drawn from each village. We performed annual cross-sectional surveys of households enrolled in the PHIRST study at both sites to assess housing conditions.

### 2.2. Study Population and Sample

Eligible households were defined as three or more people who regularly shared at least four meals together per week. Households were eligible for inclusion in the study if they had been residing in the study sites for at least one year prior to the start of the study. Over the three-year period, data were collected on the characteristics of the main dwelling for a total of 326 residential sites: 159 in Agincourt and 167 in Jouberton. The response rates in Agincourt and Jouberton, respectively, equaled 100% and 99%. The study protocol was registered on clinicaltrials.gov on 6 August 2015 and was approved by the University of the Witwatersrand’s Human Research Ethics Committee (HREC certificate number 150808). The U.S. Centers for Disease Control and Prevention’s Institutional Review Board relied on the local review (CDC IRB #6840).

### 2.3. Data Collection and Methods

Using a pre-structured questionnaire and an observation sheet, trained field workers collected data on living conditions using hand-held digital devices and Research Electronic Data Capture (REDCap) tools [17]. Respondents, classified as household members aged 18 years or older, were asked about socio-demographic information, housing type and condition, sources and storage of water, fuels used for cooking and space heating (and associated costs), mechanisms for solid waste disposal, presence of ceilings, and condition of local roads. Water sources were categorized as low risk (indoor tap); moderate risk (outdoor or off-site tap); or high risk (borehole, river, dam, stream, water vendor, water truck, water tank). Interviewers collected information on dwelling construction materials, thermal comfort devices used during hot weather and vegetative covering of yards/gardens through direct observation. Participants provided individual written consent or assent prior to enrolment and received a grocery store voucher of R 25–30 (USD$ 2–2.5) per visit as compensation for participation.

### 2.4. Data Analysis

The collected data were downloaded from the REDCap database, and cleaned and analyzed with STATA statistical software (version 15, StataCorp LLC., College Station, TX, USA). Data were analyzed and tabulated using absolute numbers and proportions for key variables in each of the two study sites. For categorical variables, the Pearson chi-square or Fishers exact test was applied to determine the relationships between the study site, year of study, living conditions, and factors potentially associated with air quality. For continuous variables, a Shapiro–Wilk test was used to determine whether the variables were normally distributed or not. A non-parametric test, two-sample Wilcoxon rank-sum test, was applied to test whether the average household monthly expenditure on electricity, paraffin, and wood differed significantly across study sites. The K-sample equality of median test was used to determine whether median values were statistically different at the 0.05 level of significance.

## 3. Results

**Figure 2:**
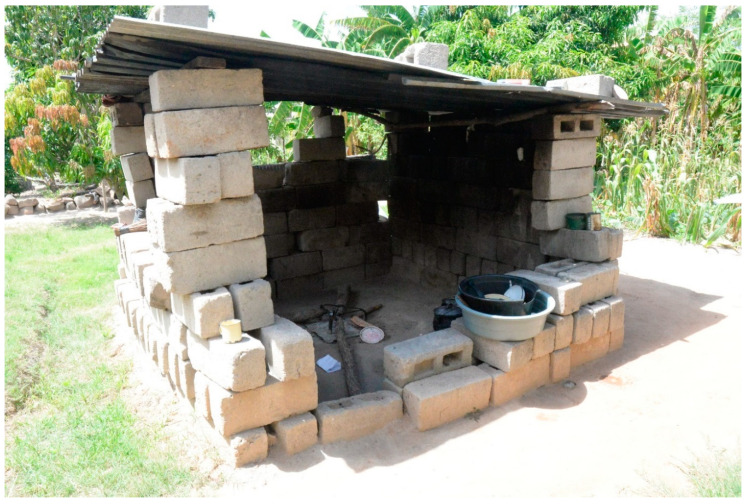
An outdoor kitchen in the Agincourt study site, South Africa: PHIRST ^†^, 2016–2018. ^†^ PHIRST: Prospective Household cohort study of Influenza and Respiratory Syncytial virus community burden and Transmission dynamics in South Africa. Photograph: Angela Mathee.

In 2016, 2017, and 2018, housing data were collected annually for 50, 53, and 56 dwellings in Agincourt and 50, 55, and 62 dwellings in Jouberton, respectively (Table 1). In both Agincourt and Jouberton, at least 80% of dwellings were brick-and-mortar structures that had been built by the occupants themselves or by a procured builder. In both study sites, corrugated metal sheets were used as roofing material in more than three-quarters of dwellings (*p* = 0.294). In more than 70% of dwellings in both Agincourt and Jouberton no ceilings (ceilings absent throughout the dwelling), or only partial ceilings (ceilings in some rooms only) were in place. The median age of dwelling structures was 15 years in both Agincourt (interquartile range [IQR]: 9–23 years) and Jouberton Township (IQR: 8–24 years) (*p* = 0.383). Dwellings in Agincourt had a median of four rooms (IQR: 2–6 rooms) (comprised of kitchens, bedrooms, lounges, and dining rooms) in the interior, and a median of one (IQR: 1–2 rooms) additional exterior room (kitchens, bathrooms, or bedrooms) located outside the primary dwelling. In Jouberton, there was similarly a median of four interior rooms (IQR: 3–5 rooms) (*p* = 0.211), but a median of zero (IQR: 0–1 rooms) additional exterior rooms in the yard (*p* < 0.001). Seventy-eight per cent of toilets in Agincourt and 45% of toilets in Jouberton were located apart from the main dwelling (*p* < 0.001). In 69% of dwellings in Agincourt and 16% in Jouberton, kitchens were rudimentary structures, also separated from the main dwelling structure (*p* < 0.001) (see example in Figure 2). In Agincourt, 41% of dwellings had a detached bathroom, which was significantly higher than Jouberton (10%) (*p* < 0.001). More than half of the study dwellings (42% in Agincourt and 57% in Jouberton) showed signs of degradation, such as cracks in walls (*p* = 0.005).

### 3.1. Water Supply

**Figure 3:**
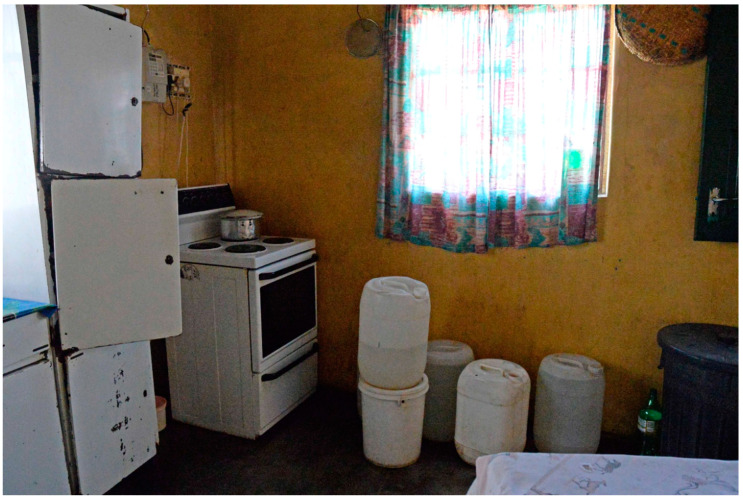
Water storage in an Agincourt kitchen, South Africa: PHIRST ^†^, 2016–2018. ^†^ PHIRST: Prospective Household cohort study of Influenza and Respiratory Syncytial virus community burden and Transmission dynamics in South Africa. Photograph: Angela Mathee.

The differences between Agincourt and Jouberton with respect to water access and security (the reliability of the water supply) were stark (Table 2). In Agincourt, 75% (119/159) of households reported daily or weekly interruptions in water supply, compared to 1% (1/167) in Jouberton. In Agincourt, 89% of respondents said that their water supply was interrupted on a monthly basis or more frequently, while in Jouberton, 87% of respondents reported that water supply interruptions hardly ever occurred (*p* < 0.001). In Agincourt, 86% of households obtained water from multiple sources (more than half of households in Agincourt used four or more sources of water) and 28% of households sourced water from a tap beyond the boundaries of their dwelling site, or from boreholes, rivers, dams, streams, water tanks, water vendors, and water delivery trucks. In Jouberton Township, all households obtained water from a tap, either indoors or on the dwelling site (*p* < 0.001). The primary water source for 22% of Agincourt households was regarded as high risk, while none of the households in Jouberton used high risk water sources (*p* < 0.001). For all water sources combined, 3% of Jouberton households used high risk water sources, compared to 89% in Agincourt (*p* < 0.001). More households in Agincourt (97%) relative to Jouberton (69%) stored water for drinking purposes in miscellaneous containers (*p* < 0.001) (see Figure 3). In Agincourt, respondents reported that water could be stored in containers for relatively long periods: 22% stored drinking water in containers for a month or more, compared with 1% in Jouberton (*p* < 0.001). Flies and other insects were reported to gain access to stored water: 26% in Agincourt and 12% in Jouberton (*p* = 0.006). Two percent of dwellings in Agincourt had running hot water, compared with 13% in Jouberton (*p* < 0.001) (Table 2).

### 3.2. Fuel Use and Perceptions of Air Quality

Overall, in both study sites, electricity was the most widely used fuel for daily cooking: 56% in Agincourt and 93% in Jouberton (*p* < 0.001). In both study sites, the use of multiple cooking fuels was widespread: 78% in Agincourt and 52% in Jouberton (*p* < 0.001). Sixty percent of Agincourt respondents reported that they did not heat their dwellings during cold weather compared to 13% in Jouberton (*p* < 0.001). In Jouberton, 75% of households used electricity to heat the indoor environment (Table 3).

Waste removal services were absent for the most part in Agincourt (97% of Agincourt households lacked waste removal services), but widely used in Jouberton (98%) (*p* < 0.001). In Agincourt, 86% of households burned their waste on a fortnightly or more frequent basis, compared with 23% in Jouberton (*p* < 0.001). The proportion of households that included a tobacco smoker was lower in Agincourt (14%) relative to Jouberton (52%) (*p* < 0.001). In both study sites, a high proportion of dwellings were located on an unpaved road (67% in Agincourt and 47% in Jouberton) (*p* = 0.003), and most respondents in Agincourt (77%) and Jouberton (91%) reported that on windy days the local air became very dusty (*p* = 0.001). More than half of the area of dwelling sites in Agincourt (68%) and Jouberton (87%) was comprised of bare soil (uncovered by vegetation) (*p* < 0.001). More respondents in Agincourt (64%) than Jouberton (33%) perceived there to be a local air pollution problem (*p* < 0.001). The most important source of air pollution reported in Agincourt was the combustion of domestic waste, while dust from unpaved roads, as well as smoke, were the main sources cited in Jouberton (Table 3). 

Within each of the two study sites, housing conditions also differed across villages (Agincourt) and samples (Jouberton). In 2016, 30% of Agincourt households used electricity for cooking while 70% used wood. By contrast in the 2018 study villages, 73% of households used electricity for cooking, with a relatively small proportion (27%) using wood. In Jouberton, electricity was used for cooking by ≥90% of the households recruited in all study years. Amongst the Agincourt study villages, for example, the differences in the proportion of households using wood as their primary cooking fuel were statistically significant (*p* < 0.001), as were differences in the type of fuel used for space heating (*p* = 0.001) and monthly expenditure on electricity (*p* = 0.015). Within Jouberton Township, there were differences across samples with respect to space heating fuel (*p* = 0.005), expenditure on wood (*p* < 0.001), frequency of household waste burning (*p* = 0.001), respondents’ perception of local air pollution (*p* = 0.001), inclusion of a smoker in households (*p* = 0.031), and the area of bare soil in the yard (*p* < 0.001).

Apart from the differences in living conditions between the two study sites, there were also differences within the study sites across the three study years. Within Agincourt, for example, there were statistically significant differences across villages with respect to location of toilets (*p* = 0.001), the absence of a ceiling (*p* = 0.003); the primary water source (*p* = 0.001), the number of different water sources used (*p* < 0.001), duration of water storage (*p* = 0.001), and frequency of water supply interruptions (*p* = 0.002). There were also differences across study samples within Jouberton in respect of type of roof (*p* = 0.002), presence of cracks in walls (*p* < 0.001), leaking water pipes (*p* = 0.032), presence of mold (*p* = 0.001), and primary water source (*p* = 0.006). The differences across villages in Agincourt and across samples in Jouberton are given in Supplementary Tables S1 (living conditions) and Table S2 (factors associated with fuel use and air quality).

## 4. Discussion

We identified multiple housing-related hazards to health in both the rural (Agincourt) and urban (Jouberton) South African study sites. Household water supplies, especially in Agincourt, presented health concerns from the perspectives of quality, access, quantity, and security. Systematic reviews have demonstrated a significant association between inadequate water (and sanitation) and elevated risks of diarrheal disease [19]. In any context other than the supply of water through an indoor tap, households are highly likely to store water in miscellaneous containers within their dwellings for convenient access, for example at night or during poor weather. Water quality is known to deteriorate substantially between source and storage containers. A meta-analysis of 45 studies undertaken in poorly resourced countries showed that the mean percentage of contaminated water samples increased from 46% at the source, to 75% in water storage containers [20]. In villages similar to Agincourt, in the neighboring province of Limpopo, bacteriological analyses indicated that 60% of water samples collected from kitchen storage containers had a total coliform count exceeding 100 counts/100 mL, which is defined as indicative of a significant and increasing risk of infectious disease transmission [21]. Furthermore, the unwieldy procedure of having to access water for handwashing from a container, may reduce levels of handwashing, which has been shown to significantly increase the risk of influenza infection [12,13,22].

Relative to Jouberton Township, there is heightened concern for water-related ill health in the Agincourt villages, especially given the findings of simultaneously widespread use of high-risk water sources, water insecurity as illustrated by a high frequency of water supply interruptions and protracted storage of water in containers. The findings may reflect urban–rural disparities in investment in basic environmental health infrastructure, such as water supply. Such concern is further escalated in the current era of climate change, with local studies demonstrating statistically significant associations between certain weather patterns and hospital admissions for diarrhea [23].

The combustion of unsafe solid and liquid fuels such as wood, coal, and paraffin for daily cooking and space heating has been associated with the emission of particulate matter, which may reach very high concentrations indoors [24]. Fine particulate matter has been shown to penetrate deeply into the lungs, where it may cause inflammation, making those exposed vulnerable to respiratory infections [25]. While proxy measures, rather than objective measurements, of pollution exposure were used for this analysis, the results nevertheless point to several potential sources of participant exposure to particulate matter or dust in the study communities. The widespread use of solid and liquid fuels for cooking and space heating, the absence of municipal waste collection services leading to frequent backyard combustion of solid waste, and unpaved roads are likely among the key sources of exposure to particulate matter. As is the case for water supply, health concerns over exposure to particulate matter from biomass [26] are highest in Agincourt, where reports of relatively widespread use of wood fuel are substantiated by high monthly household expenditure on wood. Tobacco use was an exception, being more prevalent in Jouberton Township relative to Agincourt, which is typical of the established urban-rural divide in tobacco use practices in South Africa [27]. This study identified additional housing factors with potential impacts on personal and community health, including degraded dwelling structures (such as leaking roofs, cracks in walls, and leaking water pipes), the potential for dust generated by vehicles driving on unpaved roads to enter dwellings, indoor dampness from leaking roofs and water pipes, and the concomitant proliferation of mold [28]. While, relative to Agincourt, the environmental health status in the Jouberton Township site was superior, there were nevertheless concerns about the proportion of dwellings with exterior toilets, absent ceilings, wall cracks, leaking roofs, unpaved roads in the neighborhood, and the perception of high levels of local airborne dust and air pollution.

While the current study sites appear to be typical of many human settlements in South Africa, unique local aspects of the built environment, as well as socio-cultural dynamics imply that the study findings should be generalized with caution. Nevertheless, this study provides in-depth characterizations of housing conditions in two settings, and housing typologies, that are generally under-studied in South Africa. The findings give an important account of the extent of environmental risks to health in the PHIRST study sites in Agincourt and Jouberton and indicate multiple pathways through which the health of the resident communities may be compromised by their housing conditions. The findings indicate a high degree of variability in the quality of living conditions across and within the study sites, indicative of potential health risk differences, which will need to be considered in further investigations of infection and transmission of influenza and other respiratory tract pathogens in the PHIRST study.

Overall, the study revealed how multiple facets of housing in the study sites fall short of the definitions of healthy housing produced by various United Nations agencies. The Office of the United Nations High Commissioner for Human Rights (UN-HABITAT) for example has stated that “housing is not adequate if its occupants do not have safe drinking water, adequate sanitation, energy for cooking, heating, lighting, food storage or refuse disposal” [29]. The WHO guidelines on housing and health state that “interruptions to drinking-water supply are a major determinant of the access to and quality of drinking-water” and that “safe water storage and handling in households is important for ensuring that treated water does not become re-contaminated” [1]. In Agincourt especially, the water supply was often unsafe, being widely obtained from risky sources, and characterized by frequent interruptions and storage in unsealed containers. Moreover, in Agincourt, the widespread, daily use of wood for cooking and space heating is likely to be contributing to elevated particulate exposure among those undertaking cooking tasks or spending time close to fires during combustion, for example young children. Daily use of solid fuels, even if mostly in outdoor kitchens, is also likely to be detrimentally affecting ambient as well as indoor air quality, since outdoor particulate matter is an important contributor to indoor air pollution, in urban as well as rural areas [30]. Given the evidence that housing and environmental factors may be associated with as much as a quarter of the total global burden of disease [2], improvements in housing conditions and basic environmental services in the current study sites and other similar settings could materially improve human health.

## 5. Conclusions

This study has revealed multiple housing-related hazards, and provided a finer understanding of the environmental and housing characteristics that may affect the health of communities in poor rural and urban settings in South Africa. The prevailing living conditions are likely to be detrimentally impacting the health of local residents, especially in vulnerable groups such as children and those with pre-existing conditions. Housing conditions across the study sites vary dramatically; these differences need to be factored into comparisons of infectious disease rates during follow-up studies to avoid confounding and misclassification biases. Analyses of the burdens of ill health attributable to water-related concerns and indoor/ambient air pollution, as well as degraded housing structures, would be of value in efforts to tackle health hazards in living environments.

## Figures and Tables

**Table 1:** Housing conditions in Agincourt and Jouberton, South Africa: PHIRST ^†^, 2016–2018.

	Agincourt				Jouberton				
Year (n)	2016 (50) n (%)	2017 (53) n (%)	2018 (56) n (%)	2016-2018 (159) N (%)	2016 (50) n (%)	2017 (55) n (%)	2018 (62) n (%)	2016–2018 (167) N (%)	* *p*-Value Test for difference between Agincourt and Jouberton (2016 to 2018)
Type of dwelling									
Bricks-and-mortar dwelling built by professional builder	32 (64%)	31 (58%)	29 (52%)	92 (58%)	39 (78%)	48 (87%)	56 (90%)	143 (86%)	<0.001
Bricks-and-mortar dwelling built by self	11 (22%)	20 (38%)	26 (46%)	57 (36%)	6 (12%)	5 (9%)	4 (6%)	15 (9%)	
Informal structure	5 (10%)	1 (2%)	0 (0%)	6 (4%)	3 (6%)	2 (4%)	1 (2%)	6 (4%)	
Other dwelling	2 (4%)	1 (2%)	1 (2%)	4 (3%)	2 (4%)	0 (0%)	1 (2%)	3 (2%)	
Age of the dwelling (years)									
1–8	8 (16%)	9 (17%)	22 (40%)	39 (25%)	21 (42%)	14 (25%)	15 (24%)	50 (30%)	0.373
9–15	14 (28%)	15 (28%)	12 (21%)	41 (26%)	10 (20%)	17 (31%)	14 (23%)	41 (25%)	
16–23	17 (34%)	13 (25%)	12 (21%)	42 (26%)	13 (26%)	10 (19%)	9 (15%)	32 (19%)	
>23	11 (22%)	16 (30%)	10 (18%)	37 (23%)	6 (12%)	14 (25%)	24 (39%)	44 (26%)	
Median (IQR)	18.5 (10–25)	16 (11–25)	10 (7–20)	15 (9–23)	10.5 (5–20)	14 (8–24)	15 (8–25)	15 (8–24)	
Dwellings with kitchen detached from main dwelling	33 (66%)	34 (64%)	43 (77%)	110 (69%)	10 (20%)	7 (13%)	9 (15%)	26 (16%)	<0.001
Dwellings with toilet located outside/separate from main dwelling	39 (78%)	36 (68%)	49 (88%)	124 (78%)	25 (50%)	23 (42%)	27 (44%)	75 (45%)	<0.001
Dwellings with bathroom located outside/separate from main dwelling	16 (32%)	20 (38%)	29 (52%)	65 (41%)	4 (8%)	6 (11%)	6 (10%)	16 (10%)	<0.001
Dwellings with corrugated metal sheet roof	42 (84%)	41 (77)	38 (68)	121 (76%)	47 (94%)	43 (78%)	44 (72%)	134 (81%)	0.294
Dwellings with no ceiling	43 (86%)	30 (57%)	43 (77)	116 (73%)	39 (78%)	40 (73%)	51 (82%)	130 (78%)	0.305
Cracks in walls	29 (58%)	18 (66%)	19 (35%)	66 (42%)	30 (60%)	43 (78%)	23 (37%)	96 (57%)	0.005
Leaking roofs	24 (48%)	19 (36%)	28 (50%)	71 (48%)	26 (52%)	28 (51%)	33 (53%)	87 (52%)	0.179
Leaking water pipes in or around dwelling	5 (10%)	3 (6%)	4 (7%)	12 (8%)	5 (10%)	16 (29%)	13 (21%)	34 (20%)	0.002
House has no ceilings	7 (14%)	23 (43%)	13 (23%)	43 (27%)	11 (22%)	15 (27%)	11 (18%)	37 (22%)	0.305
Fungus or mold on walls or ceiling	7 (14%)	8 (15%)	6 (11%)	21 (13%)	3 (6%)	6 (11%)	1 (2%)	10 (6%)	0.232

^†^ PHIRST: Prospective Household cohort study of Influenza and Respiratory Syncytial virus community burden and Transmission dynamics in South Africa. * *p*-Value of the χ2 or Fisher’s exact or Wilcoxon rank-sum test of association within study sites (significant at *p* < 0.05).

**Table 2:** Access to and risks related to water supply in Agincourt and Jouberton, South Africa: PHIRST ^†^, 2016–2018.

	Agincourt				Jouberton				
Year (n)	2016 (50) n (%)	2017 (53) n (%)	2018 (56) n (%)	2016–2018 (159) N (%)	2016 (50) n (%)	2017 (55) n (%)	2018 (62) n (%)	2016–018 (167) N (%)	* *p*-Value Test for difference between Agincourt and Jouberton (2016 to 2018)
Primary water source:									
Indoor tap	27 (54%)	47 (89%)	8 (14%)	82 (52%)	16 (32%)	34 (62%)	32 (52%)	82 (49%)	<0.001
Tap in yard	10 (20%)	3 (6%)	18 (32%)	31 (20%)	34 (68%)	21 (38%)	30 (48%)	85 (51%)	
Off-site tap	7 (14%)	2 (4%)	2 (4%)	11 (7%)	0 (0%)	0 (0%)	0 (0%)	0 (0%)	
Water tank	0 (0%)	1 (2%)	9 (16%)	10 (6%)	0 (0%)	0 (0%)	0 (0%)	0 (0%)	
Water vendor or truck	3 (6%)	0 (0%)	3 (5%)	6 (4%)	0 (0%)	0 (0%)	0 (0%)	0 (0%)	
Borehole	3 (6%)	0 (0%)	13 (23%)	16 (10%)	0 (0%)	0 (0%)	0 (0%)	0 (0%)	
River or stream or dam	0 (0%)	0 (0%)	1 (2%)	1 (1%)	0 (0%)	0 (0%)	0 (0%)	0 (0%)	
Number of different water sources:									
1	4 (8%)	1 (2%)	17 (30%)	22 (14%)	37 (74%)	39 (71%)	36 (58%)	112 (67%)	<0.001
2	7 (14%)	3 (6%)	20 (36%)	30 (19%)	13 (26%)	14 (25%)	25 (40%)	52 (31%)	
3	11 (22%)	3 (6%)	10 (18%)	24 (15%)	0 (0%)	2 (4%)	1 (2%)	3 (2%)	
>4	28 (56%)	46 (87%)	9 (16%)	83 (52%)	0 (0%)	0 (0%)	0 (0%)	0 (0%)	
Level of water risk [18]:									
Low risk (makes use of indoor tap only)	2 (4%)	1 (2%)	1 (2%)	4 (3%)	4 (8%)	23 (42%)	8 (13%)	35 (21%)	<0.001
Moderate risk (outdoor tap, on or off-site)	2 (4%)	3 (6%)	8 (14%)	13 (8%)	45 (90%)	28 (51%)	54 (87%)	127 (76%)	
High risk (may use borehole, river, dam, stream, water vendor, water tank, or water truck)	46 (92%)	49 (92%)	47 (84%)	142 (89%)	1 (2%)	4 (7%)	0 (0%)	5 (3%)	
Drinking water is stored in a container	47 (94%)	53 (100%)	54 (100%)	154 (97%)	38 (76%)	38 (69%)	39 (64%)	115 (69%)	<0.001
How long is water stored in a container?									
1 day or less	4 (9%)	16 (30%)	26 (50%)	46 (30%)	30 (79%)	10 (26%)	29 (78%)	69 (61%)	<0.001
2–7 days	9 (19%)	11 (21%)	8 (15%)	28 (18%)	7 (18%)	28 (74%)	8 (22%)	43 (38%)	
8 to 30 days	20 (43%)	12 (23%)	13(25%)	45 (29%)	1 (3%)	0 (0%)	0 (0%)	1 (1%)	
>30 days	14 (30%)	14 (26%)	6 (11%)	34 (22%)	0 (0%)	0 (0%)	0 (0%)	0 (0%)	
Flies and other insects sometimes get into the drinking water container	11 (23%)	13 (25%)	16 (30%)	40 (26%)	6 (16%)	5 (13%)	3 (8%)	14 (12%)	0.006
Frequency of water supply interruptions:									
Never/hardly ever/infrequently	2 (4%)	14 (26%)	1 (2%)	17 (11%)	36 (72%)	48 (87%)	61 (98%)	145 (87%)	<0.001
Monthly	10 (20%)	7 (13%)	5 (9%)	22 (14%)	13 (26%)	7 (13%)	1 (2%)	21 (13%)	
Weekly	27 (54%)	30 (57%)	8 (15%)	65 (41%)	1 (2%)	0 (0%)	0 (0%)	1 (1%)	
Daily	11 (22%)	2 (4%)	41 (75%)	54 (34%)	0 (0%)	0 (0%)	0 (0%)	0 (0%)	
Access to running hot water	0 (0%)	3 (6%)	0 (0%)	3 (2%)	3 (6%)	9 (16%)	9 (15%)	21 (13%)	<0.001
Type of toilet:									
Waterborne flush toilet	0 (0%)	5 (9%)	0 (0%)	5 (3%)	50 (100%)	55 (100%)	59 (97%)	164 (99%)	<0.001
Pit latrine	48 (96%)	46 (87%)	53 (96%)	147 (93%)	0 (0%)	0 (0%)	2 (3%)	2 (1%)	
No toilet (make use of bush)	2 (4%)	2 (4%)	2 (4%)	6 (4%)	0 (0%)	0 (0%)	0 (0%)	0 (0%)	

^†^ PHIRST: Prospective Household cohort study of Influenza and Respiratory Syncytial virus community burden and Transmission dynamics in South Africa. * *p*-Value of the χ2 or Fisher’s exact or Wilcoxon rank-sum test of association within study sites (significant at *p* < 0.05).

**Table 3:** Factors potentially associated with air quality in Agincourt and Jouberton Township, South Africa: PHIRST ^†^, 2016–2018.

Study Site	Agincourt				Jouberton				^ *p*-Value Test for Difference between Agincourt and Jouberton Township
Year (n)	2016 (50)	2017 (53)	2018 (56)	2016–2018 (159)	2016 (50)	2017 (55)	2018 (62)	2016–2018 (167)	
Main fuel used for cooking:									
Electricity	15 (30%)	33 (62%)	41 (73%)	89 (56%)	45 (90%)	52 (95%)	58 (95%)	155 (93%)	<0.001
Gas	0 (0%)	0 (0%)	0 (0%)	0 (0%)	2 (4%)	1 (2%)	1 (2%)	4 (2%)	
Paraffin	0 (0%)	0 (0%)	0 (0%)	0 (0%)	3 (6%)	1 (2%)	2 (3%)	6 (4%)	
Wood	35 (70%)	20 (38%)	15 (27%)	70 (44%)	0 (0%)	1 (2%)	0 (0%)	1 (1%)	
A secondary fuel is sometimes used for cooking	36 (72%)	38 (72%)	49 (89%)	123 (78%)	32 (64%)	25 (45%)	30 (48%)	87 (52%)	<0.001
Main fuel used for space heating:									
Don’t heat the dwelling	36 (72%)	28 (53%)	30 (54%)	95 (60%)	12 (24%)	0 (0%)	9 (16%)	21 (13%)	<0.001
Electricity	6 (12%)	14 (26%)	24 (43%)	44 (28%)	29 (58%)	47 (85%)	45 (80%)	121 (75%)	
Gas	0 (0%)	0 (0%)	0 (0%)	0 (0%)	1 (2%)	1 (2%)	1 (2%)	3 (2%)	
Paraffin	0 (0%)	1 (2%)	0 (0%)	1 (1%)	4 (8%)	1 (2%)	1 (2%)	6 (4%)	
Solid fuel (wood or coal)	7 (14%)	10 (19%)	2 (4%)	19 (12%)	4 (8%)	6 (11%)	0 (0%)	10 (6%)	
A secondary fuel is sometimes used for space heating	3 (6%)	2 (4%)	10 (18%)	15 (9%)	8 (16%)	10 (18%)	5 (8%)	23 (14%)	0.215
Fuel used to heat water for personal hygiene:									
Nothing (do not heat water)	24 (48%)	27 (51%)	32 (58%)	83 (53%)	2 (4%)	0 (0%)	10 (17%)	12 (7%)	<0.001
Electricity/solar	12 (24%)	16 (30%)	15 (27%)	43 (27%)	43 (86%)	52 (95%)	45 (76%)	137 (84%)	
Gas	0 (0%)	0 (0%)	0 (0%)	0 (0%)	2 (4%)	1 (2%)	1 (2%)	4 (2%)	
Paraffin	0 (0%)	0 (0%)	1 (2%)	1 (1%)	3 (6%)	2 (4%)	3 (5%)	8 (5%)	
Solid fuel (wood or coal)	14 (28%)	10 (30%)	6 (11%)	30 (19%)	0 (0%)	0 (0%)	0 (0%)	0 (0%)	
Monthly household electricity expenditure (Rands): ^~^									
< R50 (USD 3)	Data not collected ^#^	2 (4%)	1 (2%)	3 (3%)	Data not collected	0 (0%)	0 (0%)	0 (0%)	<0.001
R50–R99 (USD 3–6)		6 (11%)	3 (5%)	9 (8%)		0 (0%)	0 (0%)	0 (0%0	
R100–R499		29 (55%)	28 (50%)	57 ((52%)		5 (9%)	9 (15%)	14 (12%)	
>R500 (USD 30)		16 (30%)	24 (43%)	40 (37%)		50 (91%)	53 (85%)	103 (88%)	
Average monthly household electricity expenditure (Rands): ~	Data not collected ^#^	x = 149	x = 209	x = 176	Data not collected	x = 365	x = 365	x = 369;	<0.001
		n = 52;	n = 54;	n = 108;		n = 55;	n = 60;	n = 115;	
		range = 20–500;	range = 50–950;	range = 0–950;		range = 100–1200;	range = 100–3000;	range = 100–3000;	
		SD = 97.14;	SD = 154.73;	SD = 133.60;		SD = 387.01;	SD = 387.01;	SD = 308.89;	
		median = 100;	median = 165;	median = 150;		median = 360;	median = 300;	median = 300;	
		IQR = 50–300	IQR = 100–400	IQR = 50–500		IQR = 150–600	IQR = 100–700	IQR = 100–900	
Monthly paraffin household expenditure (Rands): ^~^									
0 Rands (USD 0)	Data not collected ^#^	48 (91%)	40 (71%)	88 (81%)	Data not collected	43 (78%)	30 (48%)	73 (62%)	< 0.001
R 10–R 100 (USD 0.6–6.0)		5 (9%)	15 (27%)	20 (18%)		8 (15%)	24 (39%)	32 (27%)	
>R 100 (USD 6.0)		0 (0%)	1 (2%)	1 (1%)		4 (7%)	8 (13%)	12 (11%)	
Average monthly household paraffin expenditure (Rands): ^~^	Data not collected ^#^	x = 35	x = 33	x = 6	Data not collected	x = 122	x = 37	x = 22	0.074
		n = 5;	n = 16;	n = 109		n = 12;	n = 25;	n = 110;	
		range = 14–60;	range = 13–130;	range = 0–130		range = 15–300	range = 12–150;	range = 0–300;	
		SD = 19.63;	SD = 29.92;	SD = 17.79		SD = 97.18	SD = 33.87;	SD = 51.81;	
		median = 30;	median = 24;	median = 0		median = 100;	median = 24;	median = 0;	
		IQR = 0–39	IQR = 17–39	IQR = 0–50		IQR = 50–150	IQR = 13–60	IQR = 0–150	
Monthly household wood expenditure (Rands): ^~^									
0 Rands (USD 0)	Data not collected ^#^	34 (64%)	28 (50%)	62 (57%)	Data not collected	49 (89%)	45 (73%)	94 (81%)	<0.001
R 1–R 200 (USD 0.06–12)		11 (21%)	10 (18%)	21 (19%)		4 (7%)	1 (2%)	5 (4%)	
>R 200 (USD 12)		8 (15%)	18 (32%)	26 (24%)		2(4%)	16 (26)	18 (15%)	
Average monthly household wood expenditure (Rands): ^~^	Data not collected ^#^	x = 249	x = 316	x = 125	Data not collected	x = 175	x = 60	x = 11	0.044
		n = 19;	n = 28;	n = 109;		n = 6;	n = 1;	n = 101;	
		range = 80–500;	range = 80–500;	range = 0–1100;		range = 50–300;	range = 60–60;	range = 0–300;	
		SD = 144.60;	SD = 225.20;	SD = 193.66;		SD = 88.03;	SD = 8.85;	SD = 46.23;	
		median = 200;	median = 275;	median = 0;		median = 150;	median = 60;	median = 0;	
		IQR = 100–450	IQR = 70–500	IQR = 0–500		IQR = 150–150	IQR = 0–0	IQR = 0–150	
The local neighborhood is not provided with waste collection services	46 (92%)	53 (100%)	56 (100%)	155 (97%)	0 (0%)	2 (4%)	2 (3%)	4 (2%)	<0.001
Household waste is sometimes burned at home	46 (92%)	43 (81%)	43 (77%)	132 (83%)	18 (36%)	20 (36%)	15 (24%)	53 (32%)	<0.001
Frequency of household waste burning (amongst those who burn waste):									
Daily	4/46 (9%)	11/43 (26%)	2/43 (5%)	17 (13%)	1/18(6%)	0/20 (0%)	0/14 (0%)	1 (2%)	<0.001
Weekly or fortnightly	34/46 (74%)	22/43 (51%)	40/43 (93%)	96 (73%)	3/18(17%)	8/20 (40%)	0/14 (0%)	11 (21%)	
Monthly	5/46 (11%)	8/43 (18%)	1/43 (2%)	14 (11%)	4/18(22%)	12/20 (60%)	4/14 (29%)	20 (38%)	
Rarely	3/46 (7%)	2/43 (5%)	0 (0%)	5 (4%)	10/18(56%)	0/20 (0%)	10/14 (71%)	30 (38%)	
Respondent perceives neighborhood air is polluted	31 (62%)	43 (81%)	28 (50%)	102 (64%)	32 (64%)	4 (7%)	19 (31%)	55 (33%)	<0.001
During windy weather the air gets very dusty	36 (72%)	43 (81%)	44 (79%)	123 (77%)	45 (90%)	54 (98%)	53 (85%)	152 (91%)	0.001
The household includes a smoker	9 (18%)	10 (19%)	3 (5%)	22 (14%)	25 (50%)	35 (64%)	27 (44%)	87 (52%)	<0.001
The household keeps pets	24 (48%)	8 (15%)	6 (11%)	38 (24%)	7 (14%)	28 (51%)	15 (24%)	50 (30%)	0.232
The household keeps animals for food generation purposes ^#^	Data not collected ^#^	15 (28%)	12 (21%)	27 (25%)	Data not collected	5 (9%)	1 (2%)	6 (5%)	<0.001
The road on which the house is located is unpaved	46 (92%)	51 (96%)	55 (98%)	106 (67%)	34 (68%)	31 (56%)	48 (77%)	79 (47%)	0.003
Area of yard/garden covered by vegetation									
<50%	35 (70%)	45 (85%)	26 (48%)	106 (68%)	47 (94%)	54 (98%)	44 (72%)	145 (87%)	<0.001
>50%	15 (30%)	8 (15%)	28 (52%)	51 (32%)	3 (6%)	1 (2%)	17 (28%)	21 (13%)	
There are shade trees on the plot	40 (80%)	47 (89%)	44 (79%)	131 (82%)	39 (78%)	41 (75%)	39 (63%)	119 (71%)	0.017

^†^ PHIRST: Prospective Household cohort study of Influenza and Respiratory Syncytial virus community burden and Transmission dynamics in South Africa. ^#^ Collection of data on selected factors commenced only in 2017. ^ *p*-Value of the χ2 or Fisher’s exact or Wilcoxon rank-sum test of association by study site. ^~^ United States Dollar:South African Rand exchange rate of 1:16.8 (12 July 2020).

## Data Availability

The investigators welcome enquiries about possible collaborations and access to the data set. Investigators interested in more details about this study, or in accessing these resources, should contact the principle investigator, Prof Cheryl Cohen, at NICD (cherylc@nicd.ac.za).

## Supplementary Materials

The following are available at https://www.mdpi.com/1660-4601/18/5/2240/s1, Table S1: Comparison of living conditions within Agincourt and Jouberton, South Africa: PHIRST^†^, 2016–2018, Table S2: Comparisons of factors potentially associated with air quality within Agincourt and Jouberton Township, South Africa: PHIRST^†^, 2016–2018.

## References

[1] World Health Organization World Health Organization Housing and Health Guidelines. Geneva, Switzerland: World Health Organization 2018. https://www.who.int/publications/i/item/who-housing-and-health-guidelines.

[2] Prüss-Ustün A., Wolf J., Corvalán C., Neville T., Bos R., Neira M. (2017). Diseases due to unhealthy environments: An updated estimate of the global burden of disease attributable to environmental determinants of health. J. Public Health.

[3] Bartram J., Cairncross S. (2010). Hygiene, sanitation, and water: Forgotten foundations of health. PLoS Med..

[4] Saunders-Hastings P., Crispo J.A., Sikora L., Krewski D. (2017). Effectiveness of personal protective measures in reducing pandemic influenza transmission: A systematic review and meta-analysis. Epidemics.

[5] Mbakaya B.C., Lee P.H., Lee R.L. (2017). Hand hygiene intervention strategies to reduce diarrhoea and respiratory infections among schoolchildren in developing countries: A systematic review. Int. J. Environ. Res. Public Health.

[6] Gordon S.B., Bruce N.G., Grigg J., Hibberd P.L., Kurmi O.P., Lam K.-B.H., Mortimer K., Asante K.P., Balakrishnan K., Balmes J. (2014). Respiratory risks from household air pollution in low- and middle-income countries. Lancet Respir. Med..

[7] World Health Organization WHO guidelines for Indoor Air Quality: Household Fuel Combustion, Geneva, Switzerland, World Health Organization 2014. https://www.who.int/airpollution/guidelines/household-fuel-combustion/en/.

[8] Quinn A.K., Bruce N., Puzzolo E., Dickinson K., Sturke R., Jack D.W., Mehta S., Shankar A., Sherr K., Rosenthal J.P. (2018). An analysis of efforts to scale up clean household energy for cooking around the world. Energy Sustain. Dev..

[9] Pope C.A., Ezzati M., Cannon J.B., Allen R.T., Jerrett M., Burnett R.T. (2018). Mortality risk and PM 2.5 air pollution in the USA: An analysis of a national prospective cohort. Air Qual. Atmos. Health.

[10] Wang B., Liu Y., Li Z., Li Z. (2016). Association of indoor air pollution from coal combustion with influenza-like illness in housewives. Environ. Pollut..

[11] Chen G., Zhang W., Li S., Zhang Y., Williams G., Huxley R., Ren H., Cao W., Guo Y. (2017). The impact of ambient fine particles on influenza transmission and the modification effects of temperature in China: A multi-city study. Environ. Int..

[12] Rabie T., Curtis V. (2006). Handwashing and risk of respiratory infections: A quantitative systematic review. Trop. Med. Int. Health.

[13] Liu M., Ou J., Zhang L., Shen X., Hong R., Ma H., Zhu B.-P., Fontaine R.E. (2016). Protective effect of hand-washing and good hygienic habits against seasonal influenza: A case-control study. Medicine.

[14] World Health Organization Guidelines for Drinking-Water Quality. Fourth ed. Geneva, Switzerland: World Health Organization 2011..

[15] Kahn K., Collinson M.A., Gómez-Olivé F.X., Mokoena O., Twine R., Mee P., Afolabi S.A., Clark B.D., Kabudula C.W., Khosa A. (2012). Profile: Agincourt health and socio-demographic surveillance system. Int. J. Epidemiol..

[16] Lebina L., Fuller N., Osoba T., Scott L., Motlhaoleng K., Rakgokong M., Abraham P., Variava E., Martinson N.A. (2016). The use of Xpert MTB/Rif for active case finding among TB contacts in North West Province, South Africa. Tuberc. Res. Treat..

[17] Harris P.A., Taylor R., Thielke R., Payne J., Gonzalez N., Conde J.G. (2009). Research electronic data capture (REDCap)—A metadata-driven methodology and workflow process for providing translational research informatics support. J. Biomed. Inform..

[18] Gundry S.W., Wright J.A., Conroy R., du Preez M., Genthe B., Moyo S., Mutisi C., Ndamba J., Potgieter N. (2006). Contamination of drinking water between source and point-of-use in rural household of South Africa and Zimbabwe: Implications for monitoring the Millenium Development Goal for water. Water Pract. Technol..

[19] Wolf J., Prüss-Ustün A., Cumming O., Bartram J., Bonjour S., Cairncross S., Clasen T., Colford J.M., Curtis V., De France J. (2014). Systematic review: Assessing the impact of drinking water and sanitation on diarrhoeal disease in low-and middle-income settings: Systematic review and meta-regression. Trop. Med. Int. Health.

[20] Shields K.F., Bain R.E., Cronk R., Wright J.A., Bartram J. (2015). Association of supply type with fecal contamination of source water and household stored drinking water in developing countries: A bivariate meta-analysis. Environ. Health Perspect..

[21] Kapwata T., Mathee A., Le Roux W.J., Wright C.Y. (2018). Diarrhoeal disease in relation to possible household risk factors in South African villages. Int. J. Environ. Res. Public Health.

[22] Luby S.P., Agboatwalla M., Painter J., Altaf A., Billhimer W.L., Hoekstra R.M. (2004). Effect of intensive handwashing promotion on childhood diarrhea in high-risk communities in Pakistan: A randomized controlled trial. JAMA.

[23] Ikeda T., Kapwata T., Behera S.K., Minakawa N., Hashizume M., Sweijd N., Mathee A., Wright C.Y. (2019). Climatic Factors in relation to diarrhoea hospital admissions in rural Limpopo, South Africa. Atmosphere.

[24] Bruce N., Pope D., Rehfuess E., Balakrishnan K., Adair-Rohani H., Dora C. (2015). WHO indoor air quality guidelines on household fuel combustion: Strategy implications of new evidence on interventions and exposure–risk functions. Atmos. Environ..

[25] Rylance J., Fullerton D.G., Scriven J., Aljurayyan A.N., Mzinza D., Barrett S., Wright A.K., Wootton D.G., Glennie S.J., Baple K. (2015). Household air pollution causes dose-dependent inflammation and altered phagocytosis in human macrophages. Am. J. Respir. Cell Mol. Biol..

[26] Bruce N.G., Dherani M.K., Das J.K., Balakrishnan K., Adair-Rohani H., Bhutta Z.A., Pope D. (2013). Control of household air pollution for child survival: Estimates for intervention impacts. BMC Public Health.

[27] Peer N., Bradshaw D., Laubscher R., Steyn N., Steyn K. (2013). Urban–rural and gender differences in tobacco and alcohol use, diet and physical activity among young black South Africans between 1998 and 2003. Glob. Health Action.

[28] Mendell M.J., Mirer A.G., Cheung K., Tong M., Douwes J. (2011). Respiratory and allergic health effects of dampness, mold, and dampness-related agents: A review of the epidemiologic evidence. Environ. Health Perspect..

[29] United Nations-Habitat The Right to Adequate Housing. Fact Sheet. Geneva, Switzerland: Office of the United Nations High Commissioner for Human Rights 2014..

[30] Snider G., Carter E., Clark S., Yang X., Ezzati M., Schauer J.J., Wiedinmyer C., Baumgartner J. (2018). Impacts of stove use patterns and outdoor air quality on household air pollution and cardiovascular mortality in southwestern China. Environ. Int..

