# Supplementary material for: Housing Quality in a Rural and an Urban Settlement in South Africa
Source: Int J Environ Res Public Health. 2021 Feb 24;18(5):2240. doi: 10.3390/ijerph18052240 (PMC7956558; doi:10.3390/ijerph18052240)
Supplement: Supplementary file 1 [file ijerph-18-02240-s001.pdf]

Supplementary Table S1. Comparison of living conditions within Agincourt and Jouberton, South Africa: PHIRST[†], 2016–2018.

	Agincourt					Jouberton				
**Year (n)**	2016 (50) n (%)	2017 (53) n (%)	2018 (56) n (%)	2016-2018 (159) N (%)	*P-value Test for the difference within Agincourt (2016 to 2018	2016 (50) n (%)	2017 (55) n (%)	2018 (62) n (%)	2016-2018 (167) N (%)	*P-value Test for the difference within Jouberton (2016 to 2018)
**Type of dwelling:**										
**Bricks-and-mortar dwelling built by professional builder**	32 (64%)	31 (58%)	29 (52%)	92 (58%)	0.070	39 (78%)	48 (87%)	56 (90%)	143 (86%)	0.359
**Bricks-and-mortar dwelling built by self**	11 (22%)	20 (38%)	26 (46%)	57 (36%)		6 (12%)	5 (9%)	4 (6%)	15 (9%)	
**Informal structure**	5 (10%)	1 (2%)	0 (0%)	6 (4%)		3 (6%)	2 (4%)	1 (2%)	6 (4%)	
**Other dwelling**	2 (4%)	1 (2%)	1 (2%)	4 (3%)		2 (4%)	0 (0%)	1 (2%)	3 (2%)	
**Age of the dwelling (years):**										
**1 – 8**	8 (16%)	9 (17%)	22 (40%)	39 (25%)	0.152	21 (42%0	14 (25%)	15 (24%)	50 (30%)	0.261
**9 – 15**	14 (28%)	15 (28%)	12 (21%)	41 (26%)		10 (20%)	17 (31%)	14 (23%)	41 (25%)	
**16 – 23**	17 (34%)	13 (25%)	12 (21%)	42 (26%)		13 (26%)	10 (19%)	9 (15%)	32 (19%)	
**>23**	11 (22%)	16 (30%)	10 (18%)	37 (23%)		6 (12%)	14 (25%)	24 (39%)	44 (26%)	
**Median (IQR)**	18.5 (10 – 25)	16 (11 – 25)	10 (7 – 20)	15 (9 – 23)		10.5 (5 – 20)	14 (8 – 24)	15 (8 – 25)	15 (8- 24)	
**Dwellings with kitchen detached from main dwelling,**	33 (66%)	34 (64%)	43 (77%)	110 (69%)	0.162	10 (20%)	7 (13%)	9 (15%)	26 (16%)	0.543
**Dwellings with toilet located outside/separate from main dwelling,**	39 (78%)	36 (68%)	49 (88%)	124 (78%)	0.001	25 (50%)	23 (42%)	27 (44%)	75 (45%)	0.704
**Dwellings with bathroom located outside/separate from main dwelling**	16 (32%)	20 (38%)	29 (52%)	65 (41%)	0.104	4 (8%)	6 (11%)	6 (10%)	16 (10%)	0.877

**Dwellings with corrugated metal sheet roof**	42 (84%)	41 (77)	38 (68)	121 (76 %)	0.147	47 (94%)	43 (78%)	44 (72%)	134 (81%)	0.002
**Dwellings with no ceiling**	43 (86%)	30 (57%)	43 (77)	116 (73%)	0.003	39 (78%)	40 (73%)	51 (82%)	130 (78%)	0.473
**Cracks in walls**	29 (58%)	18 (66%)	19 (35%)	66 (42%)	0.363	30 (60%)	43 (78%)	23 (37%)	96 (57%)	<0.001
**Leaking roofs**	24 (48%)	19 (36%)	28 (50%)	71 (48%)	0.276	26 (52%)	28 (51%)	33 (53%)	87 (52%)	0.967
**Leaking water pipes in or around dwelling**	5 (10%)	3 (6%)	4 (7%)	12 (8%)	0.720	5 (10%)	16 (29%)	13 (21%)	34 (20%)	0.032
**House has no ceilings**	7 (14%)	23 (43%)	13 (23%)	43 (27%)	0.135	11 (22%)	15 (27%)	11 (18%)	37 (22%)	0.001
**Fungus or mould on walls or ceiling**	7 (14%)	8 (15%)	6 (11%)	21 (13%)	0.239	3 (6%)	6 (11%)	1 (2%)	10 (6%)	0.006
**Primary water source:**										
**Indoor tap**	27 (54%)	47 (89%)	8 (14%)	82 (52%)		16 (32%)	34 (62%)	32 (52%)	82 (49%)	
**Tap in yard**	10 (20%)	3 (6%)	18 (32%)	31 (20%)		34 (68%)	21 (38%)	30 (48%)	85 (51%)	
**Off-site tap**	7 (14%)	2 (4%)	2 (4%)	11 (7%)		0 (0%)	0 (0%)	0 (0%)	0 (0%)	
**Water tank**	0 (0%)	1 (2%)	9 (16%)	10 (6%)	<0.001	0 (0%)	0 (0%)	0 (0%)	0 (0%)	0.006
**Water vendor or truck**	3 (6%)	0 (0%)	3 (5%)	6 (4%)		0 (0%)	0 (0%)	0 (0%)	0 (0%)	
**Borehole**	3 (6%)	0 (0%)	13 (23%)	16 (10%)		0 (0%)	0 (0%)	0 (0%)	0 (0%)	
**River or stream or dam**	0 (0%)	0 (0%)	1 (2%)	1 (1%)		0 (0%)	0 (0%)	0 (0%)	0 (0%)	
**Number of different water sources:**										
**1**	4 (8%)	1 (2%)	17 (30%)	22 (14%)		37 (74%)	39 (71%)	36 (58%)	112 (67%)	
**2**	7 (14%)	3 (6%)	20 (36%)	30 (19%)	<0.001	13 (26%)	14 (25%)	25 (40%)	52 (31%)	0.049
**3**	11 (22%)	3 (6%)	10 (18%)	24 (15%)		0 (0%)	2 (4%)	1 (2%)	3 (2%)	
**≥4**	28 (56%)	46 (87%)	9 (16%)	83 (52%)		0 (0%)	0 (0%)	0 (0%)	0 (0%)	
**Level of water risk:**										
**Low risk (makes use of indoor tap only)**	2 (4%)	1 (2%)	1 (2%)	4 (3%)		4 (8%)	23 (42%)	8 (13%)	35 (21%)	
**Moderate risk (outdoor tap, on or off-site)**	2 (4%)	3 (6%)	8 (14%)	13 (8%)	0.337	45 (90%)	28 (51%)	54 (87%)	127 (76%)	0.076
**High risk (may use borehole, river, dam, stream, water vendor, water tank or water truck)**	46 (92%)	49 (92%)	47 (84%)	142 (89%)		1 (2%)	4 (7%)	0 (0%)	5 (3%)	

**Drinking water is stored in a container**	47 (94%)	53 (100%)	54 (100%)	154 (97%)	0.079	38 (76%)	38 (69%)	39 (64%)	115 (69%)	0.389
**How long is water stored in a container?**										
**1 day or less**	4 (9%)	16 (30%)	26 (50%)	46 (30%)		30 (79%)	10 (26%)	29 (78%)	69 (61%)	
**2 -7 days**	9 (19%)	11 (21%)	8 (15%)	28 (18%)	0.001	7 (18%)	28 (74%)	8 (22%)	43 (38%)	<0.001
**8 to 30 days**	20 (43%)	12 (23%)	13(25%)	45 (29%)		1 (3%)	0 (0%)	0 (0%)	1 (1%)	
**>30 days**	14 (30%)	14 (26%)	6 (11%)	34 (22%)		0 (0%)	0 (0%)	0 (0%)	0 (0%)	
**Flies and other insects sometimes get into the drinking water container**	11 (23%)	13 (25%)	16 (30%)	40 (26%)	0.754	6 (16%)	5 (13%)	3 (8%)	14 (12%)	0.543
**Frequency of water supply interruptions:**										
**Never/hardly ever/infrequently**	2 (4%)	14 (26%)	1 (2%)	17 (11%)	0.003	36 (72%)	48 (87%)	61 (98%)	145 (87%)	<0.001
**Monthly**	10 (20%)	7 (13%)	5 (9%)	22 (14%)		13 (26%)	7 (13%)	1 (2%)	21 (13%)	
**Weekly**	27 (54%)	30 (57%)	8 (15%)	65 (41%)		1 (2%)	0 (0%)	0 (0%)	1 (1%)	
**Daily**	11 (22%)	2 (4%)	41 (75%)	54 (34%)		0 (0%)	0 (0%)	0 (0%)	0 (0%)	
**Access to running hot water**	0 (0%)	3 (6 %)	0 (0%)	3 (2%)	0.078	3 (6%)	9 (16%)	9 (15%)	21 (13%)	0.131
**Type of toilet:**										
**Waterborne flush toilet**	0 (0%)	5 (9%)	0 (0%)	5 (3%)	0.002	50(100%)	55 (100%)	59 (97%)	164 (99%)	0.156
**Pit latrine**	48 (96%)	46 (87%)	53 (96%)	147 (93%)		0 (0%)	0 (0%)	2 (3%)	2 (1%)	
**No toilet (make use of bush)**	2 (4%)	2 (4%)	2 (4%)	6 (4%)		0 (0%)	0 (0%)	0 (0%)	0 (0%)	

†PHIRST: Prospective Household cohort study of Influenza and Respiratory Syncytial virus community burden and Transmission dynamics in South Africa.

*P-value of the $\chi 2$ or Fisher's exact or Wilcoxon rank-sum test of association within study sites (significant at $P \leq 0.05$).

Supplementary Table S2. Comparisons of factors potentially associated with air quality within Agincourt and Jouberton Township, South Africa: PHIRST†, 2016–2018.

Study site	Agincourt					Jouberton				
**Year (n)**	2016 (50)	2017 (53)	2018 (56)	2016-2018(159)	*P-value Test for the difference within Agincourt (2016 to 2018	2016 (50)	2017 (55)	2018 (62)	2016-2018 (167)	*P-value Test for the difference within Jouberton (2016 to 2018
**Main fuel used for cooking:**										
**Electricity**	15 (30%)	33 (62%)	41 (73%)	89 (56%)		45 (90%)	52 (95%)	58 (95%)	155 (93%)	
**Gas**	0 (0%)	0 (0%)	0 (0%)	0 (0%)		2 (4%)	1 (2%)	1 (2%)	4 (2%)	
**Paraffin**	0 (0%)	0 (0%)	0 (0%)	0 (0%)	<0.001	3 (6%)	1 (2%)	2 (3%)	6 (4%)	0.593
**Wood**	35 (70%)	20 (38%)	15 (27%)	70 (44%)		0 (0%)	1 (2%)	0 (0%)	1 (1%)	
**A secondary fuel is sometimes used for cooking**	36 (72%)	38 (72%)	49 (89%)	123 (78%)	0.022	32 (64%)	25 (45%)	30 (48%)	87 (52%)	0.119
**Main fuel used for space heating:**										
**Don't heat the dwelling**	36 (72%)	28 (53%)	30 (54%)	95 (60%)		12 (24%)	0 (0%)	9 (16%)	21 (13%)	
**Electricity**	6 (12%)	14 (26%)	24 (43%)	44 (28%)		29 (58%)	47 (85%)	45 (80%)	121 (75%)	
**Gas**	0 (0%)	0 (0%)	0 (0%)	0 (0%)	0.001	1 (2%)	1 (2%)	1 (2%)	3 (2%)	0.005
**Paraffin**	0 (0%)	1 (2%)	0 (0%)	1 (1%)		4 (8%)	1 (2%)	1 (2%)	6 (4%)	
**Solid fuel (wood or coal)**	7 (14%)	10 (19%)	2 (4%)	19 (12%)		4 (8%)	6 (11%)	0 (0%)	10 (6%)	
**A secondary fuel is sometimes used for space heating**	3 (6%)	2 (4%)	10 (18%)	15 (9%)	0.055	8 (16%)	10 (18%)	5 (8%)	23 (14%)	0.216
**Fuel used to heat water for personal hygiene:**										
**Nothing (do not heat water)**	24 (48%)	27 (51%)	32 (58%)	83 (53%)		2 (4%)	0 (0%)	10 (17%)	12 (7%)	0.065
**Electricity/solar**	12 (24%)	16 (30%)	15 (27%)	43 (27%)	0.778	43 (86%)	52 (95%)	45 (76%)	137 (84%)	
**Gas**	0 (0%)	0 (0%)	0 (0%)	0 (0%)		2 (4%)	1 (2%)	1 (2%)	4 (2%)	
**Paraffin**	0 (0%)	0 (0%)	1 (2%)	1 (1%)		3 (6%)	2 (4%)	3 (5%)	8 (5%)	
**Solid fuel (wood or coal)**	14 (28%)	10 (30%)	6 (11%)	30 (19%)		0 (0%)	0 (0%)	0 (0%)	0 (0%)	
**Monthly household electricity expenditure (Rands):~**										

**< R50 (USD 3)**<br>**R50 - R99 (USD 3 – 6)**<br>**R100 - R499**<br>**> R500 (USD 30)**	Data not collected	2 (4%)<br>6 (11%)<br>29 (55%)<br>16 (30%)	1 (2%)<br>3 (5%)<br>28 (50%)<br>24 (43%)	3 (3%)<br>9 (8%)<br>57 ((52%)<br>40 (37%)	0.266	Data not collected	0 (0%)<br>0 (0%)<br>5 (9%)<br>50 (91%)	0 (0%)<br>0 (0%)<br>9 (15%)<br>53 (85%)	0 (0%)<br>0 (0%0<br>14 (12%)<br>103 (88%)	0.365
**Average monthly household electricity expenditure (Rands):~**	Data not collected	x̄=149<br>n=52;<br>range=20-500;<br>SD=97.14;<br>median=100;<br>IQR=50-300	x̄=209<br>n=54;<br>range=50-950;<br>SD=154.73;<br>median=165;<br>IQR=100-400	x̄=176<br>n=108;<br>range=0-950<br>SD=133.60<br>median=150<br>IQR=50-500	0.015	Data not collected	x̄=365<br>n=55;<br>range=100-1200;<br>SD=387.01;<br>median=360;<br>IQR=150-600	x̄=365<br>n=60;<br>range=100-3000;<br>SD=387.01;<br>median=300;<br>IQR=100-700	x̄=369;<br>n=115;<br>range=100-3000;<br>SD=308.89;<br>median=300;<br>IQR=100-900	0.094
**Monthly paraffin household expenditure (Rands):~**<br>**0 Rands (USD 0)**<br>**R 10 – R 100 (USD 0.6 – 6.0)**<br>**> R 100 (USD 6.0)**	Data not collected	48 (91%)<br>5 (9%)<br>0 (0%)	40 (71%)<br>15 (27%)<br>1 (2%)	88 (81%)<br>20 (18%)<br>1 (1%)	0.319	Data not collected	43 (78%)<br>8 (15%)<br>4 (7%)	30 (48%)<br>24 (39%)<br>8 (13%)	73 (62%)<br>32 (27%)<br>12 (11%)	0.313
**Average monthly household paraffin expenditure (Rands):~**	Data not collected	x̄=35<br>n=5;<br>range=14-60;<br>SD=19.63;<br>median=30;<br>IQR=0-39	x̄=33<br>n=16;<br>range=13-130;<br>SD=29.92;<br>median=24;<br>IQR=17-39	x̄=6<br>n=109<br>range=0-130<br>SD=17.79<br>median=0<br>IQR=0-50	0.015	Data not collected	x̄=122<br>n=12;<br>range=15-300<br>SD=97.18<br>median=100;<br>IQR=50-150	x̄=37<br>n=25;<br>range=12-150;<br>SD=33.87;<br>median=24;<br>IQR=13-60	x̄=22<br>n=110;<br>range=0-300;<br>SD=51.81;<br>median=0;<br>IQR=0-150	0.074
**Monthly household wood expenditure (Rands):~**<br>**0 Rands (USD 0)**<br>**R 1 – R 200 (USD 0.06 - 12)**<br>**> R 200 (USD 12)**	Data not collected	34 (64%)<br>11 (21%)<br>8 (15%)	28 (50%)<br>10 (18%)<br>18 (32%)	62 (57%)<br>21 (19%)<br>26 (24%)	<0.001	Data not collected	49 (89%)<br>4 (7%)<br>2(4%)	45 (73%)<br>1 (2%)<br>16 (26)	94 (81%)<br>5 (4%)<br>18 (15%)	< 0.001
**Average monthly household wood expenditure (Rands):~**	Data not collected	x̄=249<br>n=19;<br>range=80-500;	x̄=316<br>n=28;<br>range=80-500;	x̄=125<br>n=109;<br>range=0-1100;<br>SD=193.66;	0.092	Data not collected	x̄=175<br>n=6;<br>range=50-300;<br>SD=88.03;	x̄=60<br>n=1;<br>range=60-60;<br>SD=8.85;	x̄=11<br>n=101;<br>range=0-300;<br>SD=46.23;	0.081

		SD=144.60; median=200; IQR=100-450	SD=225.20; median=275; IQR=70-500	median=0; IQR=0-500			median=150; IQR=150-150	median=60; IQR=0-0	median=0; IQR=0-150	
**The local neighbourhood is not provided with waste collection services**	46 (92%)	53 (100%)	56 (100%)	155 (97%)	0.041	0 (0%)	2 (4%)	2 (3%)	4 (2%)	0.134
**Household waste is sometimes burned at home**	46 (92%)	43 (81%)	43 (77%)	132 (83%)	0.058	18 (36%)	20 (36%)	15 (24%)	53 (32%)	0.257
**Frequency of household waste burning (amongst those who burn waste):**<br>**Daily**<br>**Weekly or fortnightly**<br>**Monthly**<br>**Rarely**	 <br><br>4 (9%)<br>34 (74%)<br>5 (11%)<br>3 (7%)	 <br><br>1 (26%)<br>22 (51%)<br>8 (18%)<br>2 (5%)	 <br><br>2 (5%)<br>40 (93%)<br>1 (2%)<br>0 (0%)	 <br><br>17 (13%)<br>96 (73%)<br>14 (11%)<br>5 (4%)	0.002	 <br><br>1 (6%)<br>3 (17%)<br>4 (22%)<br>10 (56%)	 <br><br>0 0%)<br>8 (40%)<br>12 (60%)<br>0 (0%)	 <br><br>0 (0%)<br>0 (0%)<br>4 (29%)<br>10 (71%)	 <br><br>1 (2%)<br>11 (21%)<br>20 (38%)<br>30 (38%)	0.001
**Respondent perceives neighbourhood air is polluted**	31 (62%)	43 (81%)	28 (50%)	102 (64%)	0.001	32 (64%)	4 (7%)	19 (31%)	55 (33%)	<0.001
**During windy weather the air gets very dusty**	36 (72%)	43 (81%)	44 (79%)	123 (77%)	0.546	45 (90%)	54 (98%)	53 (85%)	152 (91%)	0.014
**The household includes a smoker**	9 (18%)	10 (19%)	3 (5%)	22 (14%)	0.031	25 (50%)	35 (64%)	27 (44%)	87 (52%)	0.850
**The household keeps pets**	24 (48%)	8 (15%)	6 (11%)	38 (24%)	0.001	7 (14%)	28 (51%)	15 (24%)	50 (30%)	0.001
**The household keeps animals for food generation purposes**[#]	Data not collected	15 (28%)	12 (21%)	27 (25%)	0.412	Data not collected	5 (9%)	1 (2%)	6 (5%)	0.080
**The road on which the house is located is unpaved**	46 (92%)	51 (96%)	55 (98%)	106 (67%)	0.553	34 (68%)	31 (56%)	48 (77%)	79 (47%)	0.019
**Area of yard/garden covered by vegetation:**<br>**<50 %**<br>**>50%**	 35 (70%)<br>15 (30%)	 45 (85%)<br>8 (15%)	 26 (48%)<br>28 (52%)	 106 (68%)<br>51 (32%)	<0.001	 47 (94%)<br>3 (6%)	 54 (98%)<br>1 (2%)	 44 (72%)<br>17 (28%)	 145 (87%)<br>21 (13%)	0.001
**There are shade trees on the plot**	40 (80%)	47 (89%)	44 (79%)	131 (82%)	0.281	39 (78%)	41 (75%)	39 (63%)	119 (71%)	0.188

†PHIRST: Prospective Household cohort study of Influenza and Respiratory Syncytial virus community burden and Transmission dynamics in South Africa.
*P-value of the $\chi 2$ or Fisher's exact or Wilcoxon rank-sum test of association within study sites (significant at $P \leq 0.05$). #Collection of data on selected factors commenced only in 2017.
